# Supplementary material for: Developing an Interpretable Machine Learning Model to Predict in-Hospital Mortality in Sepsis Patients: A Retrospective Temporal Validation Study
Source: J Clin Med. 2023 Jan 24;12(3):915. doi: 10.3390/jcm12030915 (PMC9917524; doi:10.3390/jcm12030915)
Supplement: Supplementary file 1 [file jcm-12-00915-s001.zip › jcm-2078880-supplementary.pdf]

Supplementary Materials

Table S1. Feature selection strategies of model development.

Features	Unit	XGBoost			LR		
		Basic + Lab	Basic + Intervention	Whole	Basic + Lab	Basic + Intervention	Whole
**Demographic characteristics**							
Age	years	×	×	×	×	×	×
Gender	%	×	×	×	×	×	×
Race	%	×	×	×	×	×	×
BMI	kg/m^2	×	×	×	×	×	×
Marital status	%	×	×	×	×	×	×
**Pre-existing diseases**							
Myocardial infarction	%	×	×	×	×	×	×
Congestive heart failure	%	×	×	×	×	×	×
Cerebrovascular disorder	%	×	×	×	×	×	×
Chronic pulmonary disease	%	×	×	×	×	×	×
Chronic kidney disease	%	×	×	×	×	×	×
Diabetes	%	×	×	×	×	×	×
Cancer	%	×	×	×	×	×	×
**Vital signs (Maximum, minimum values)**							
Heart rate	bpm	×	×	×	×	×	×
Systolic blood pressure	mmHg	×	×	×	×	×	×
Diastolic blood pressure	mmHg	×	×	×	×	×	×
Mean blood pressure	mmHg	×	×	×	×	×	×
Respiratory rate	bpm	×	×	×	×	×	×
Temperature	℃	×	×	×	×	×	×
Peripheral oxygen saturation	%	×	×	×	×	×	×
**Laboratory measurements (Maximum, minimum values)**							
White blood cell	10^9/L	×		×	×		×
Hematocrit	%	×		×	×		×
Hemoglobin	g/dL	×		×	×		×
Platelets	10^9/L	×		×	×		×
Mean corpuscular hemoglobin	pg	×		×	×		×
Mean corpuscular hemoglobin concentration	g/dL	×		×	×		×
Mean corpuscular volume	fl	×		×	×		×
Red blood cell	10^{12}/L	×		×	×		×

Red blood cell distribution width	%	×		×	×		×
Neutrophil-to-lymphocyte ratio		×		×	×		×
Glucose	mmol/L	×		×	×		×
Blood urea nitrogen	mg/dL	×		×	×		×
Creatinine	mg/dL	×		×	×		×
Bicarbonate	mmol/L	×		×	×		×
Sodium	mmol/L	×		×	×		×
Potassium	mmol/L	×		×	×		×
Chloride	mmol/L	×		×	×		×
Calcium	mg/dL	×		×	×		×
Anion gap	mmol/L	×		×	×		×
Albumin	g/dL	×		×	×		×
Alanine transaminase	IU/L	×		×	×		×
Alkaline phosphatase	IU/L	×		×	×		×
Aspartate transaminase	IU/L	×		×	×		×
Total bilirubin	mg/dL	×		×	×		×
Lactate dehydrogenase	IU/L	×		×	×		×
Creatine kinase (myocardial band)	ng/mL	×		×	×		×
Prothrombin time	seconds	×		×	×		×
Partial thromboplastin time	seconds	×		×	×		×
International normalized ratio		×		×	×		×
Fibrinogen	mg/dL	×		×	×		×
**Blood gas results (Maximum, minimum values)**							
Lactate	mmol/L	×		×	×		×
pH		×		×	×		×
PaO_2	mmHg	×		×	×		×
$PaCO_2$	mmHg	×		×	×		×
Oxygenation index	mmHg	×		×	×		×
Base Excess	mmol/L	×		×	×		×
Total CO_2	mmol/L	×		×	×		×
**Clinical interventions**							
Invasive ventilation	%		×	×		×	×
Non-invasive ventilation	%		×	×		×	×
High flow nasal cannula	%		×	×		×	×
Supplemental Oxygen	%		×	×		×	×
Renal replacement therapy	%		×	×		×	×
Dobutamine	%		×	×		×	×

Maximum dobutamine rate	mcg/kg/min	×	×	×	×
Dopamine	%	×	×	×	×
Maximum dopamine rate	mcg/kg/min	×	×	×	×
Epinephrine	%	×	×	×	×
Maximum epinephrine rate	mcg/kg/min	×	×	×	×
Norepinephrine	%	×	×	×	×
Maximum norepinephrine rate	mcg/kg/min	×	×	×	×
Phenylephrine	%	×	×	×	×
Maximum phenylephrine rate	mcg/kg/min	×	×	×	×
Vasopressin	%	×	×	×	×
Maximum vasopressin rate	mcg/kg/min	×	×	×	×
Parenteral nutrition	%	×	×	×	×
Enteral nutrition	%	×	×	×	×
Calorie intake	Kcal	×	×	×	×
Protein intake	g	×	×	×	×
Insulin amount	IU	×	×	×	×
Total intravenous input	mL	×	×	×	×
Urine output	mL	×	×	×	×
Glasgow Coma Scale		×	×	×	×

XGBoost, extreme gradient boosting; LR, logistic regression.

Table S2. Variables and cut-off values that were selected in severity scores utilized in the study.

Variables	SAPS-II	APS-III	LODS	OASIS	SOFA
Age, years	<40; <60; <70; <75; <80; >=80			<24; <=53; <=77; <90; >=90	
Heart rate, bpm	<40; <70; <120; <160; >=160	<40; <50; <100; <110; <120; <140; <155; >=155	<30; <140; >=140	<33; <89; <=106; <=125; >125	
Systolic BP, mmHg	<70; <100; <200; >=200		<40; <70; <90; <240; <270; >=270		
Mean BP, mmHg		<40; <60; <70; <80; <100; <120; <130; <140; >=140		<20.65; <51; <61.33; <=143.44; >143.44	<70; >=70
Temperature, ℃	>=39.0	<33.0; <33.5; <34.0; <35.0; <36.0; <40.0; >=40.0		<33.22; <=35.93; <=36.39; <=36.88; <=39.88; >39.88	
Respiratory rate, bpm		<6; <12; <14; <25; <35; <40; <50; >=50		<6; <13; <=22; <=30; <=44; >44	
PaO_2/FiO_2	<100; <200; >=200		<150; >=150		<100; <200; <300; <400; >=400
PaO_2, mmHg		<50; <70; <80; >=80			
AaDO2, mmHg		<100; <250; <350; <500; >=500			
pH		<7.20; <7.30; <7.35; <7.45; <7.50; <7.60; >=7.60			
PaCO2, mmHg		<25; <30; <35; <40; <45; <50; >=50			
Blood urea nitrogen, mg/dL	<28; <84; >=84	<17.0; <20.0; <40.0; <80.0; >=80.0	<7.50; <17.0; <28.0; <56.0; >=56.0		
Creatinine, mg/dL		<0.5; <1.5; <1.95; >=1.95	<1.20; <1.60; >=1.60		<1.2; <2.0; <3.5; <5.0; >=5.0

White blood cell, 10³/mm³	<1.0; <20.0; >=20.0	<1.0; <3.0; <20.0; <25.0; >=25.0	<1.0; <2.5; <50.0; >=50.0		
Platelets, 10³/mm³			<50.0; >=50.0		<20; <50; <100; <150; >=150
Hematocrit, %		<41.0; <50.0; >=50.0			
Potassium, mEq/L	<3.0; <5.0; >=5.0				
Sodium, mEq/L	<125; <145; >=145	<120; <135; <155; >=155			
Bicarbonate, mEq/L	<15.0; <20.0; >=20.0				
Bilirubin, mg/dL	<4.0; <6.0; >=6.0	<2.0; <3.0; <5.0; <8.0; >=8.0	<2.0; >=2.0		<1.2; <2.0; <6.0; <12.0; >=12.0
Albumin, g/dL		<2.0; <2.5; <4.5; >=4.5			
Glucose, mmol/L		<40; <60; <200; <350; >=350			
Prothrombin time, seconds			<3; <15; >=15		
Urine output, mL	<500; <1000; >=1000	<400; <600; <900; <1500; <2000; <4000; >=4000	<500; <750; <10000; >=10000	<671.09; <=1426.99; <=2544.14; <=6896.80; >6896.80	<200; <500; >=500
Glasgow Coma Scale	<6; <9; <11; <14; >=14	Separate scoring according to eyes, verbal, and motor subscores	<=5; <=8; <=13; >13	<=7; <14; 14; 15	<6; <10; <13; <=14; 15
Chronic disease	AIDS; Hematologic malignancy; Metastatic cancer				
Type of admission	Scheduled surgical; Medical; Unscheduled surgical				
Ventilation				Yes; No	Yes; No
Elective surgery				Yes; No	
Pre-ICU LOS, minutes				<10.2; <297; <1440; <18708; >=18708	

Vasopressor rate, mcg/kg/min	Dopamine (0; <=5; <=15; >15); Epinephrine/Norepinephrine (0; <=0.1; >0.1); Dobutamine (0; >0)

SOFA, sequential organ failure assessment; SAPS, simplified acute physiology score; LODS, logistic organ dysfunction score; OASIS, oxford acute severity of illness score.

Table S3. The ranges of hyperparameter tuning for XGBoost models.

Hyperparameters	Ranges	Final values		
		Basic + Lab	Basic + Intervention	Whole
eta	0.01~0.3	0.07	0.21	0.01
max_depth	6~10	7	7	7
gamma	0~5	0.61	0.35	0.58
subsample	0.6~0.9	0.7	0.72	0.63
colsample_by_tree	0.5~0.8	0.72	0.63	0.51
min_child_weight	1~40	35	15	26
nround	1~5000	153	208	1458
lambda	10~50	17.89	41.9	25.5
seed.number	1~1000	222	643	828

XGBoost, extreme gradient boosting; eta, learning rate; max_depth, maximum depth of a tree; gamma, minimum loss reduction to make a further leaf node; subsample, subsample proportion; colsample_by_tree, subsample ratio of columns when constructing a tree; min_child_weight, minimum sum of instance weight needed in a child node; nround, iterations; lambda, L2 regularization; seed.number, randomized seed number.

Table S4. Vital signs, laboratory measurements, and clinical interventions of 24,272 sepsis patients stratified by in-hospital mortality.

Features	Total (N=24,272)	Survivors (N=20,513)	Non-survivors (N=3,759)	P-value
**Vital signs**				
Minimum heart rate, Mean (SD)	71.9 (15.4)	71.4 (15.0)	74.6 (17.6)	< 0.001
Maximum heart rate, Mean (SD)	106.3 (21.1)	105.2 (20.4)	112.2 (23.4)	< 0.001
Minimum systolic BP, Mean (SD)	88.5 (15.9)	89.3 (15.3)	84.1 (18.1)	< 0.001
Maximum systolic BP, Mean (SD)	147.3 (22.5)	147.5 (22.2)	145.8 (24.3)	< 0.001
Minimum diastolic BP, Mean (SD)	44.7 (10.5)	45.1 (10.2)	42.7 (11.6)	< 0.001
Maximum diastolic BP, Mean (SD)	86.9 (20.0)	86.6 (19.7)	88.2 (21.6)	< 0.001
Minimum mean BP, Mean (SD)	57.2 (11.8)	57.8 (11.5)	54.4 (13.1)	< 0.001
Maximum mean BP, Mean (SD)	104.2 (22.0)	104.0 (21.5)	104.9 (24.3)	0.362
Minimum respiratory rate, Mean (SD)	12.9 (3.4)	12.7 (3.3)	13.9 (3.8)	< 0.001
Maximum respiratory rate, Mean (SD)	28.6 (6.6)	28.2 (6.5)	30.3 (6.9)	< 0.001
Minimum temperature, Median (Q1, Q3)	36.4 (36.0, 36.7)	36.4 (36.0, 36.7)	36.4 (35.8, 36.6)	
Maximum temperature, Median (Q1, Q3)	37.4 (37.0, 37.9)	37.4 (37.0, 37.9)	37.3 (36.9, 37.9)	< 0.001
Minimum SpO2, Median (Q1, Q3)	93.0 (90.0, 95.0)	93.0 (90.0, 95.0)	92.0 (88.0, 94.0)	< 0.001
Maximum SpO2, Median (Q1, Q3)	100.0 (100.0, 100.0)	100.0 (100.0, 100.0)	100.0 (100.0, 100.0)	< 0.001
**Laboratory results**				
Minimum WBC, Median (Q1, Q3)	9.8 (6.9, 13.3)	9.6 (6.9, 13.0)	10.7 (7.0, 15.1)	< 0.001
Maximum WBC, Median (Q1, Q3)	13.9 (10.0, 18.8)	13.7 (9.9, 18.4)	15.0 (10.2, 20.8)	< 0.001
Minimum hematocrit, Mean (SD)	29.6 (6.4)	29.6 (6.3)	29.4 (6.8)	0.002
Maximum hematocrit, Mean (SD)	34.7 (6.2)	34.7 (6.1)	34.4 (6.9)	< 0.001
Minimum hemoglobin, Mean (SD)	9.8 (2.1)	9.9 (2.1)	9.6 (2.3)	< 0.001

Maximum hemoglobin, Mean (SD)	11.4 (2.1)	11.4 (2.1)	11.2 (2.3)	< 0.001
Minimum platelets, Median (Q1, Q3)	178.3 (101.7)	179.4 (99.1)	172.4 (115.0)	< 0.001
Maximum platelets, Median (Q1, Q3)	221.8 (113.4)	222.1 (110.1)	219.9 (129.9)	< 0.001
Minimum MCH, Mean (SD)	29.8 (2.6)	29.8 (2.5)	29.9 (2.9)	0.267
Maximum MCH, Mean (SD)	30.4 (2.7)	30.4 (2.6)	30.6 (3.0)	< 0.001
Minimum MCHC, Mean (SD)	32.6 (1.7)	32.7 (1.6)	32.0 (1.8)	< 0.001
Maximum MCHC, Mean (SD)	33.5 (1.7)	33.6 (1.7)	33.1 (1.8)	< 0.001
Minimum MCV, Mean (SD)	90.3 (7.0)	90.0 (6.8)	91.6 (7.9)	< 0.001
Maximum MCV, Mean (SD)	92.2 (7.3)	91.8 (7.0)	94.3 (8.5)	< 0.001
Minimum RBC, Mean (SD)	3.3 (0.7)	3.3 (0.7)	3.2 (0.8)	< 0.001
Maximum RBC, Mean (SD)	3.7 (0.7)	3.7 (0.7)	3.6 (0.8)	< 0.001
Minimum RDW, Median (Q1, Q3)	14.5 (13.4, 15.9)	14.3 (13.4, 15.7)	15.3 (14.1, 17.3)	< 0.001
Maximum RDW, Median (Q1, Q3)	14.9 (13.8, 16.6)	14.7 (13.7, 16.3)	16.0 (14.5, 18.2)	< 0.001
Minimum NLR, Median (Q1, Q3)	8.0 (4.5, 14.7)	7.5 (4.3, 13.6)	10.7 (5.7, 20.1)	< 0.001
Maximum NLR, Median (Q1, Q3)	9.0 (4.9, 17.1)	8.3 (4.7, 15.7)	12.9 (7.0, 25.0)	< 0.001
Minimum BUN, Median (Q1, Q3)	18.0 (13.0, 31.0)	18.0 (12.0, 28.0)	27.0 (17.0, 44.0)	< 0.001
Maximum BUN, Median (Q1, Q3)	22.0 (15.0, 37.0)	21.0 (15.0, 34.0)	33.0 (21.0, 53.0)	< 0.001
Minimum creatinine, Median (Q1, Q3)	0.9 (0.7, 1.4)	0.9 (0.7, 1.3)	1.2 (0.8, 2.0)	< 0.001
Maximum creatinine, Median (Q1, Q3)	1.1 (0.8, 1.8)	1.1 (0.8, 1.6)	1.5 (1.0, 2.5)	< 0.001
Minimum bicarbonate, Mean (SD)	21.2 (4.9)	21.6 (4.7)	19.4 (5.9)	< 0.001
Maximum bicarbonate, Mean (SD)	24.3 (4.4)	24.5 (4.2)	23.2 (5.3)	< 0.001
Minimum sodium, Median (Q1, Q3)	137.0 (134.0, 140.0)	137.0 (134.0, 140.0)	137.0 (133.0, 140.0)	< 0.001

Maximum sodium, Median (Q1, Q3)	140.0 (137.0, 143.0)	140.0 (137.0, 142.0)	140.0 (137.0, 144.0)	0.001
Minimum potassium, Mean (SD)	3.9 (0.6)	3.9 (0.6)	3.9 (0.7)	0.634
Maximum potassium, Mean (SD)	4.6 (0.9)	4.6 (0.9)	4.8 (1.0)	< 0.001
Minimum chloride, Median (Q1, Q3)	103.0 (98.0, 106.0)	103.0 (99.0, 106.0)	101.0 (96.0, 105.0)	< 0.001
Maximum chloride, Median (Q1, Q3)	107.0 (103.0, 110.0)	107.0 (103.0, 110.0)	106.0 (101.0, 110.0)	< 0.001
Minimum calcium, Median (Q1, Q3)	8.0 (7.5, 8.5)	8.0 (7.5, 8.5)	7.9 (7.3, 8.5)	< 0.001
Maximum calcium, Median (Q1, Q3)	8.5 (8.0, 9.0)	8.5 (8.0, 9.0)	8.5 (8.0, 9.1)	< 0.001
Minimum AG, Median (Q1, Q3)	13.0 (11.0, 15.0)	12.0 (10.0, 15.0)	14.0 (12.0, 17.0)	< 0.001
Maximum AG, Median (Q1, Q3)	16.0 (13.0, 19.0)	15.0 (13.0, 18.0)	18.0 (15.0, 22.0)	< 0.001
Minimum albumin, Mean (SD)	3.1 (0.7)	3.1 (0.7)	2.9 (0.7)	< 0.001
Maximum albumin, Mean (SD)	3.2 (0.7)	3.2 (0.7)	3.0 (0.7)	< 0.001
Minimum ALT, Median (Q1, Q3)	27.0 (16.0, 58.0)	26.0 (16.0, 54.5)	32.0 (17.0, 73.0)	< 0.001
Maximum ALT, Median (Q1, Q3)	30.0 (17.0, 69.0)	28.0 (17.0, 62.0)	37.0 (20.0, 97.5)	< 0.001
Minimum ALP, Median (Q1, Q3)	80.0 (58.0, 119.0)	77.0 (57.0, 114.0)	91.0 (63.0, 139.0)	< 0.001
Maximum ALP, Median (Q1, Q3)	87.0 (63.0, 133.0)	84.0 (61.0, 126.0)	102.0 (71.0, 160.0)	< 0.001
Minimum AST, Median (Q1, Q3)	40.0 (24.0, 85.0)	37.0 (23.0, 77.0)	51.0 (28.0, 122.0)	< 0.001
Maximum AST, Median (Q1, Q3)	45.0 (26.0, 107.0)	42.0 (25.0, 94.0)	62.0 (31.0, 165.8)	< 0.001
Minimum total bilirubin, Median (Q1, Q3)	0.7 (0.4, 1.4)	0.6 (0.4, 1.3)	0.8 (0.4, 2.0)	< 0.001
Maximum total bilirubin, Median (Q1, Q3)	0.8 (0.4, 1.7)	0.7 (0.4, 1.6)	1.0 (0.5, 2.5)	< 0.001
Minimum LDH, Median (Q1, Q3)	282.0 (207.0, 440.0)	266.0 (200.0, 394.0)	368.0 (248.0, 650.8)	< 0.001
Maximum LDH, Median (Q1, Q3)	302.0 (219.0, 490.0)	283.0 (209.0, 434.0)	409.5 (267.0, 788.2)	< 0.001
Minimum glucose, Median (Q1, Q3)	112.0 (94.0, 134.0)	112.0 (95.0, 133.0)	114.0 (91.0, 142.0)	0.295

Maximum glucose, Median (Q1, Q3)	145.0 (118.0, 192.0)	142.0 (117.0, 185.0)	165.0 (128.2, 226.0)	< 0.001
Minimum CK-MB, Median (Q1, Q3)	5.0 (3.0, 11.0)	5.0 (3.0, 10.0)	6.0 (3.0, 17.0)	< 0.001
Maximum CK-MB, Median (Q1, Q3)	6.0 (3.0, 17.0)	6.0 (3.0, 14.0)	8.0 (4.0, 26.0)	< 0.001
Minimum PT, Median (Q1, Q3)	13.5 (12.2, 15.4)	13.4 (12.1, 15.1)	14.5 (12.6, 18.4)	< 0.001
Maximum PT, Median (Q1, Q3)	14.9 (13.1, 18.0)	14.8 (13.0, 17.4)	16.5 (13.7, 23.6)	< 0.001
Minimum PTT, Median (Q1, Q3)	29.0 (25.9, 33.5)	28.7 (25.8, 32.9)	31.0 (26.7, 38.0)	< 0.001
Maximum PTT, Median (Q1, Q3)	33.2 (28.5, 44.3)	32.7 (28.4, 41.9)	38.3 (30.0, 59.9)	< 0.001
Minimum INR, Median (Q1, Q3)	1.2 (1.1, 1.4)	1.2 (1.1, 1.4)	1.3 (1.1, 1.7)	< 0.001
Maximum INR, Median (Q1, Q3)	1.4 (1.2, 1.7)	1.3 (1.2, 1.6)	1.5 (1.2, 2.2)	< 0.001
Minimum fibrinogen, Median (Q1, Q3)	220.5 (163.0, 327.0)	219.0 (165.0, 316.0)	237.0 (142.8, 373.8)	0.181
Maximum fibrinogen, Median (Q1, Q3)	240.0 (182.0, 351.0)	236.0 (181.0, 339.0)	271.0 (183.0, 413.2)	< 0.001
**Blood gas results**				
Minimum lactate, Median (Q1, Q3)	1.4 (1.0, 1.9)	1.3 (1.0, 1.8)	1.8 (1.2, 2.7)	< 0.001
Maximum lactate, Median (Q1, Q3)	2.3 (1.5, 3.6)	2.2 (1.5, 3.4)	3.0 (1.7, 5.8)	< 0.001
Minimum pH, Median (Q1, Q3)	7.3 (7.2, 7.4)	7.3 (7.3, 7.4)	7.3 (7.2, 7.4)	< 0.001
Maximum pH, Median (Q1, Q3)	7.4 (7.4, 7.5)	7.4 (7.4, 7.5)	7.4 (7.4, 7.5)	< 0.001
Minimum BE, Mean (SD)	-3.6 (5.8)	-3.1 (5.3)	-5.6 (7.5)	< 0.001
Maximum BE, Mean (SD)	0.7 (4.4)	0.9 (4.1)	-0.6 (5.5)	< 0.001
Minimum TCO2, Mean (SD)	22.5 (5.7)	22.8 (5.4)	21.0 (6.8)	< 0.001
Maximum TCO2, Mean (SD)	26.8 (5.4)	27.0 (5.1)	25.8 (6.5)	< 0.001
Minimum PaO2, Median (Q1, Q3)	91.0 (72.0, 122.0)	93.0 (74.0, 125.0)	81.0 (65.0, 108.0)	< 0.001
Maximum PaO2, Median (Q1, Q3)	237.0 (126.0, 394.0)	262.0 (133.0, 404.0)	168.0 (107.0, 296.5)	< 0.001
Minimum PaCO2, Median (Q1, Q3)	35.0 (31.0, 40.0)	35.0 (31.0, 40.0)	33.0 (28.0, 39.0)	< 0.001

Maximum PaCO2, Median (Q1, Q3)	45.0 (39.0, 52.0)	46.0 (40.0, 52.0)	44.0 (37.0, 53.0)	< 0.001
Minimum OI, Median (Q1, Q3)	195.0 (127.0, 279.0)	200.0 (134.0, 282.5)	164.0 (100.0, 257.8)	< 0.001
Maximum OI, Median (Q1, Q3)	317.5 (230.0, 412.0)	322.0 (236.7, 413.6)	290.0 (190.0, 398.0)	< 0.001
**Clinical interventions**				
RRT, %	770 (3.2%)	534 (2.6%)	236 (6.3%)	< 0.001
Invasive ventilation, %	9979 (41.1%)	8169 (39.8%)	1810 (48.2%)	< 0.001
Non-invasive ventilation, %	429 (1.8%)	349 (1.7%)	80 (2.1%)	0.068
Supplemental oxygen, %	12254 (50.5%)	10925 (53.3%)	1329 (35.4%)	< 0.001
High flow nasal cannula, %	322 (1.3%)	236 (1.2%)	86 (2.3%)	< 0.001
Dobutamine usage, %	339 (1.4%)	207 (1.0%)	132 (3.5%)	< 0.001
Maximum dobutamine rate, Median (Q1, Q3)	5.0 (2.5, 7.0)	5.0 (2.5, 5.1)	5.0 (2.5, 7.5)	0.123
Dopamine usage, %	750 (3.1%)	510 (2.5%)	240 (6.4%)	< 0.001
Maximum dopamine rate, Median (Q1, Q3)	10.0 (5.0, 15.0)	8.0 (5.0, 15.0)	10.0 (5.0, 20.0)	< 0.001
Epinephrine usage, %	1466 (6.0%)	1176 (5.7%)	290 (7.7%)	< 0.001
Maximum epinephrine rate, Median (Q1, Q3)	0.0 (0.0, 0.1)	0.0 (0.0, 0.1)	0.1 (0.1, 0.2)	< 0.001
Norepinephrine usage, %	6092 (25.1%)	4462 (21.8%)	1630 (43.4%)	< 0.001
Maximum norepinephrine rate, Median (Q1, Q3)	0.2 (0.1, 0.3)	0.2 (0.1, 0.3)	0.3 (0.1, 0.5)	< 0.001
Phenylephrine usage, %	6135 (25.3%)	5290 (25.8%)	845 (22.5%)	< 0.001
Maximum phenylephrine rate, Median (Q1, Q3)	1.0 (0.6, 2.0)	1.0 (0.5, 2.0)	2.2 (1.2, 4.1)	< 0.001
Vasopressin usage, %	1631 (6.7%)	963 (4.7%)	668 (17.8%)	< 0.001
Maximum vasopressin rate, Median (Q1, Q3)	2.4 (2.4, 2.4)	2.4 (2.4, 2.4)	2.4 (2.4, 2.4)	0.088
Parenteral nutrition, %	211 (0.9%)	151 (0.7%)	60 (1.6%)	< 0.001
Enteral nutrition, %	1215 (5.0%)	933 (4.5%)	282 (7.5%)	< 0.001
Calorie intake, Median (Q1, Q3)	178.5 (68.0, 388.4)	173.9 (68.0, 375.6)	211.7 (84.5, 454.2)	< 0.001
Protein intake, Mean (SD)	1.0 (6.2)	0.9 (5.9)	1.5 (7.7)	< 0.001
Total intravenous input, Median (Q1, Q3)	5009.9 (2742.0, 7887.9)	5103.6 (2777.9, 7886.1)	4537.8 (2572.4, 7900.6)	< 0.001
Insulin amount, Median (Q1, Q3)	0.0 (0.0, 12.0)	0.0 (0.0, 16.0)	0.0 (0.0, 6.0)	< 0.001

Urine output, Median (Q1, Q3)	1580.0 (960.0, 2395.0)	1665.0 (1065.0, 2475.0)	1010.0 (476.0, 1795.0)	< 0.001
Minimum GCS, Median (Q1, Q3)	15.0 (13.0, 15.0)	15.0 (13.0, 15.0)	15.0 (12.0, 15.0)	0.003

BP, blood pressure; SpO2, peripheral oxygen saturation; WBC, white blood cell; MCH, mean corpuscular hemoglobin; MCHC, mean corpuscular hemoglobin concentration; MCV, mean corpuscular volume; RBC, red blood cell; RDW, red blood cell distribution width; NLR, neutrophil-to-lymphocyte ratio; BUN, blood urea nitrogen; AG, anion gap; ALT, alanine transaminase; ALP, alkaline phosphatase; AST, aspartate transaminase; LDH, lactate dehydrogenase; CK-MB, creatine kinase (myocardial band); PT, prothrombin time; PTT, partial thromboplastin time; INR, international normalized ratio; PaO2, partial arterial pressure of oxygen; PaCO2, partial arterial pressure of carbon dioxide; BE, base excess; TCO2, total carbon dioxide; GCS, Glasgow Coma Scale; RRT, Renal Replacement Therapy.

Table S5. Odds ratios of multivariate logistic regression models for in-hospital mortality in the training cohort (N = 16,208).

Features	Basic + Lab				Basic + Intervention				Whole			
	ORs	2.5% CI	97.5% CI	P-value	ORs	2.5% CI	97.5% CI	P-value	ORs	2.5% CI	97.5% CI	P-value
**Baseline characteristics**												
Male (Female as reference)	0.94	0.84	1.05	0.252	1.07	0.96	1.18	0.214	1.03	0.92	1.15	0.593
Age	1.01	1.01	1.02	<0.001	1.01	1.01	1.02	<0.001	1.01	1.01	1.02	<0.001
Race (Other/Unknown as reference)												
Asian	0.67	0.48	0.94	0.019	0.69	0.49	0.96	0.028	0.67	0.48	0.95	0.023
African	0.65	0.52	0.82	<0.001	0.68	0.55	0.84	<0.001	0.64	0.51	0.80	<0.001
Hispanic	0.75	0.55	1.03	0.075	0.81	0.60	1.10	0.184	0.80	0.58	1.10	0.176
Caucasian	0.69	0.59	0.80	<0.001	0.70	0.60	0.81	<0.001	0.71	0.61	0.83	<0.001
BMI	0.98	0.98	0.99	<0.001	0.99	0.99	1.00	0.060	0.99	0.98	1.00	0.002
Marital status (Other as reference)												
Divorced/Widowed	0.45	0.36	0.56	<0.001	0.50	0.40	0.61	<0.001	0.48	0.38	0.60	<0.001
Married	0.45	0.37	0.55	<0.001	0.48	0.39	0.59	<0.001	0.47	0.38	0.58	<0.001
Single	0.46	0.37	0.57	<0.001	0.51	0.41	0.62	<0.001	0.48	0.38	0.60	<0.001
MI	1.07	0.93	1.22	0.338	1.10	0.97	1.25	0.152	1.09	0.95	1.25	0.222
CHF	0.85	0.75	0.96	0.008	0.99	0.88	1.12	0.892	0.86	0.76	0.97	0.018
CVD	2.26	1.97	2.58	<0.001	1.98	1.74	2.26	<0.001	2.08	1.82	2.39	<0.001
CPD	1.06	0.95	1.19	0.283	0.97	0.87	1.09	0.635	1.03	0.92	1.16	0.617
CKD	0.97	0.85	1.11	0.684	1.19	1.06	1.35	0.004	0.99	0.86	1.14	0.937
Diabetes	0.85	0.75	0.96	0.010	1.16	1.03	1.30	0.015	0.99	0.87	1.13	0.867
Cancer	2.03	1.79	2.31	<0.001	2.33	2.07	2.62	<0.001	2.09	1.84	2.38	<0.001
**Vital signs**												
Minimum heart rate	1.00	1.00	1.00	0.849	1.01	1.00	1.01	<0.001	1.00	1.00	1.01	0.058

Maximum heart rate	1.01	1.00	1.01	<0.001	1.01	1.01	1.01	<0.001	1.01	1.00	1.01	<0.001
Minimum systolic BP	0.99	0.99	1.00	<0.001	1.00	0.99	1.00	0.496	1.00	0.99	1.00	0.473
Maximum systolic BP	1.00	1.00	1.01	0.163	1.00	0.99	1.00	0.087	1.00	1.00	1.00	0.628
Minimum diastolic BP	1.00	0.99	1.01	0.669	1.00	0.99	1.00	0.233	1.00	0.99	1.01	0.696
Maximum diastolic BP	1.00	0.99	1.00	0.189	1.00	1.00	1.01	0.478	1.00	0.99	1.00	0.515
Minimum mean BP	1.00	0.99	1.00	0.230	1.00	0.99	1.00	0.337	1.00	0.99	1.01	0.520
Maximum mean BP	1.00	1.00	1.00	0.957	1.00	1.00	1.00	0.429	1.00	1.00	1.00	0.533
Minimum respiratory rate	1.05	1.03	1.07	<0.001	1.06	1.05	1.08	<0.001	1.05	1.03	1.07	<0.001
Maximum respiratory rate	1.01	1.00	1.02	0.009	1.02	1.01	1.03	<0.001	1.01	1.01	1.02	0.001
Minimum temperature	0.75	0.69	0.81	<0.001	0.70	0.65	0.75	<0.001	0.72	0.67	0.78	<0.001
Maximum temperature	0.99	0.92	1.06	0.689	0.81	0.76	0.87	<0.001	0.93	0.87	1.00	0.045
Minimum SpO2	0.98	0.97	0.99	<0.001	0.97	0.96	0.98	<0.001	0.98	0.97	0.99	<0.001
Maximum SpO2	0.98	0.93	1.03	0.368	0.93	0.89	0.98	0.004	0.95	0.90	1.00	0.049
**Laboratory measurements**												
Minimum WBC	1.03	1.02	1.05	<0.001	-	-	-	-	1.04	1.02	1.05	<0.001
Maximum WBC	0.98	0.97	0.99	<0.001	-	-	-	-	0.98	0.97	0.99	<0.001
Minimum hematocrit	1.04	0.99	1.10	0.101	-	-	-	-	1.03	0.98	1.09	0.236
Maximum hematocrit	0.95	0.90	0.99	0.026	-	-	-	-	0.95	0.91	1.00	0.055
Minimum hemoglobin	0.91	0.78	1.06	0.230	-	-	-	-	0.91	0.77	1.06	0.224
Maximum hemoglobin	1.10	0.95	1.28	0.198	-	-	-	-	1.10	0.94	1.28	0.240
Minimum platelets	1.00	1.00	1.00	0.699	-	-	-	-	1.00	1.00	1.00	0.553
Maximum platelets	1.00	1.00	1.00	0.911	-	-	-	-	1.00	1.00	1.00	0.280
Maximum MCH	0.93	0.80	1.07	0.303	-	-	-	-	0.93	0.80	1.08	0.361
Minimum MCH	0.86	0.73	1.01	0.066	-	-	-	-	0.86	0.73	1.02	0.083
Maximum MCHC	1.22	1.07	1.40	0.004	-	-	-	-	1.24	1.07	1.42	0.003
Minimum MCHC	0.93	0.80	1.08	0.342	-	-	-	-	0.94	0.81	1.10	0.448

Maximum MCV	1.03	0.99	1.08	0.172	-	-	-	-	1.02	0.97	1.08	0.357
Minimum MCV	1.08	1.02	1.13	0.004	-	-	-	-	1.08	1.02	1.14	0.005
Maximum RBC	0.91	0.74	1.13	0.401	-	-	-	-	0.93	0.75	1.15	0.509
Minimum RBC	1.39	1.13	1.72	0.002	-	-	-	-	1.34	1.08	1.66	0.009
Maximum RDW	1.17	1.10	1.25	<0.001	-	-	-	-	1.16	1.08	1.24	<0.001
Minimum RDW	0.97	0.91	1.05	0.472	-	-	-	-	0.97	0.90	1.04	0.426
Minimum BUN	1.02	1.01	1.03	<0.001	-	-	-	-	1.01	1.00	1.03	0.010
Maximum BUN	0.99	0.98	1.00	0.166	-	-	-	-	1.00	0.99	1.01	0.508
Minimum creatinine	1.02	0.86	1.20	0.841	-	-	-	-	1.03	0.87	1.23	0.737
Maximum creatinine	0.91	0.79	1.04	0.168	-	-	-	-	0.89	0.77	1.03	0.127
Minimum bicarbonate	0.98	0.95	1.01	0.104	-	-	-	-	0.98	0.95	1.01	0.191
Maximum bicarbonate	0.97	0.94	1.00	0.050	-	-	-	-	0.98	0.95	1.01	0.190
Minimum sodium	1.01	0.99	1.04	0.394	-	-	-	-	1.01	0.98	1.03	0.583
Maximum sodium	1.04	1.02	1.07	0.002	-	-	-	-	1.04	1.01	1.07	0.005
Minimum potassium	1.05	0.94	1.18	0.349	-	-	-	-	1.04	0.93	1.17	0.479
Maximum potassium	1.06	0.99	1.14	0.098	-	-	-	-	1.07	1.00	1.16	0.060
Minimum chloride	0.96	0.94	0.99	0.003	-	-	-	-	0.95	0.93	0.98	<0.001
Maximum chloride	0.98	0.95	1.00	0.066	-	-	-	-	1.00	0.98	1.03	0.971
Minimum calcium	0.84	0.77	0.92	<0.001	-	-	-	-	0.92	0.84	1.00	0.059
Maximum calcium	1.10	1.02	1.18	0.010	-	-	-	-	1.08	1.00	1.16	0.051
Minimum AG	1.01	0.98	1.04	0.454	-	-	-	-	1.01	0.99	1.04	0.341
Maximum AG	0.99	0.97	1.01	0.434	-	-	-	-	0.99	0.96	1.01	0.263
Minimum ALT	1.00	1.00	1.00	0.451	-	-	-	-	1.00	1.00	1.00	0.922
Maximum ALT	1.00	1.00	1.00	0.294	-	-	-	-	1.00	1.00	1.00	0.151
Minimum ALP	1.00	1.00	1.00	0.535	-	-	-	-	1.00	1.00	1.00	0.492
Maximum ALP	1.00	1.00	1.00	0.305	-	-	-	-	1.00	1.00	1.00	0.245

Minimum AST	1.00	1.00	1.00	0.954	-	-	-	-	1.00	1.00	1.00	0.858
Maximum AST	1.00	1.00	1.00	0.120	-	-	-	-	1.00	1.00	1.00	0.102
Minimum total bilirubin	1.05	0.98	1.12	0.150	-	-	-	-	1.05	0.98	1.13	0.153
Maximum total bilirubin	0.97	0.91	1.04	0.403	-	-	-	-	0.97	0.91	1.04	0.380
Minimum glucose	1.00	1.00	1.00	0.008	-	-	-	-	1.00	1.00	1.00	0.003
Maximum glucose	1.00	1.00	1.00	0.312	-	-	-	-	1.00	1.00	1.00	0.135
Minimum PT	1.00	0.97	1.04	0.776	-	-	-	-	1.00	0.97	1.03	0.962
Maximum PT	1.00	0.99	1.01	0.756	-	-	-	-	1.00	0.99	1.01	0.916
Minimum PTT	1.00	1.00	1.01	0.385	-	-	-	-	1.00	1.00	1.01	0.546
Maximum PTT	1.00	1.00	1.01	<0.001	-	-	-	-	1.00	1.00	1.01	<0.001
Minimum INR	0.98	0.71	1.35	0.898	-	-	-	-	1.04	0.74	1.46	0.829
Maximum INR	1.06	0.97	1.17	0.197	-	-	-	-	1.04	0.95	1.15	0.394
Minimum lactate	1.10	1.04	1.16	0.001	-	-	-	-	1.08	1.02	1.14	0.009
Maximum lactate	1.08	1.04	1.12	<0.001	-	-	-	-	1.08	1.04	1.12	<0.001
Minimum pH	0.28	0.08	1.02	0.054	-	-	-	-	0.47	0.12	1.80	0.272
Maximum pH	2.04	0.46	9.05	0.348	-	-	-	-	1.98	0.43	9.11	0.382
Minimum BE	1.05	1.01	1.10	0.022	-	-	-	-	1.04	1.00	1.09	0.062
Maximum BE	0.94	0.90	0.98	0.003	-	-	-	-	0.95	0.91	0.99	0.024
Minimum TCO2	0.98	0.95	1.02	0.408	-	-	-	-	0.99	0.95	1.03	0.577
Maximum TCO2	1.06	1.03	1.10	<0.001	-	-	-	-	1.05	1.01	1.09	0.011
Minimum PaO2	1.00	1.00	1.00	0.856	-	-	-	-	1.00	1.00	1.00	0.811
Maximum PaO2	1.00	1.00	1.00	<0.001	-	-	-	-	1.00	1.00	1.00	0.020
Minimum PaCO2	0.98	0.97	0.99	0.002	-	-	-	-	0.99	0.97	1.00	0.040
Maximum PaCO2	1.01	1.00	1.01	0.173	-	-	-	-	1.00	0.99	1.01	0.477
**Clinical interventions**												
Urine output	-	-	-	-	1.00	1.00	1.00	<0.001	1.00	1.00	1.00	<0.001

GCS	-	-	-	-	0.96	0.94	0.97	<0.001	0.96	0.95	0.98	<0.001
RRT	-	-	-	-	0.98	0.76	1.28	0.897	0.82	0.59	1.13	0.221
Invasive ventilation	-	-	-	-	1.28	1.14	1.44	<0.001	1.37	1.21	1.56	<0.001
Non-invasive ventilation	-	-	-	-	1.17	0.78	1.74	0.441	1.06	0.70	1.60	0.789
Supplemental oxygen	-	-	-	-	0.57	0.51	0.64	<0.001	0.61	0.54	0.68	<0.001
High flow nasal cannula	-	-	-	-	1.72	1.13	2.61	0.011	1.40	0.90	2.17	0.134
Dopamine usage	-	-	-	-	1.03	0.57	1.84	0.934	0.81	0.44	1.48	0.490
Maximum dopamine rate	-	-	-	-	1.10	1.01	1.19	0.021	1.10	1.01	1.19	0.034
Epinephrine usage	-	-	-	-	1.47	1.02	2.13	0.039	1.45	0.99	2.11	0.054
Maximum epinephrine rate	-	-	-	-	1.01	0.98	1.04	0.432	1.01	0.98	1.03	0.637
Norepinephrine usage	-	-	-	-	0.96	0.73	1.26	0.766	1.08	0.81	1.43	0.594
Maximum norepinephrine rate	-	-	-	-	2.00	0.72	5.56	0.185	1.11	0.84	1.45	0.466
Phenylephrine usage	-	-	-	-	1.48	1.30	1.68	<0.001	1.25	1.09	1.43	0.002
Maximum phenylephrine rate	-	-	-	-	1.01	0.94	1.08	0.814	0.99	0.91	1.08	0.855
Vasopressin usage	-	-	-	-	0.98	0.85	1.12	0.754	1.07	0.92	1.25	0.367
Maximum vasopressin rate	-	-	-	-	1.03	1.00	1.05	0.014	1.02	1.00	1.04	0.035
Dopamine usage	-	-	-	-	2.19	1.51	3.19	<0.001	1.63	1.18	2.24	0.003
Maximum dopamine rate	-	-	-	-	0.99	0.87	1.12	0.832	1.01	0.92	1.11	0.876
Parenteral nutrition	-	-	-	-	2.30	1.54	3.45	<0.001	2.19	1.43	3.35	<0.001
Enteral nutrition	-	-	-	-	1.38	1.08	1.76	0.010	1.30	1.01	1.67	0.046
Calorie intake	-	-	-	-	1.00	1.00	1.00	0.005	1.00	1.00	1.00	0.795
Protein intake	-	-	-	-	1.00	0.99	1.01	0.637	1.00	0.99	1.01	0.426
Total intravenous input	-	-	-	-	1.00	1.00	1.00	<0.001	1.00	1.00	1.00	<0.001
Insulin amount	-	-	-	-	0.98	0.98	0.99	<0.001	0.99	0.98	0.99	<0.001

OR, odds ratio; CI, confidence interval; MI, Myocardial infarction; CHF, Congestive Heart Failure; CVD, Cerebrovascular Disorder; CPD, Chronic Pulmonary Disease; CKD, Chronic Kidney Disease; BP, blood pressure; SpO2, peripheral oxygen saturation; WBC, white blood cell; MCH, mean corpuscular hemoglobin; MCHC, mean corpuscular hemoglobin concentration; MCV, mean corpuscular volume; RBC, red blood cell; RDW, red

blood cell distribution width; BUN, blood urea nitrogen; AG, anion gap; ALT, alanine transaminase; ALP, alkaline phosphatase; AST, aspartate transaminase; PT, prothrombin time; PTT, partial thromboplastin time; INR, international normalized ratio; PaO2, partial arterial pressure of oxygen; PaCO2, partial arterial pressure of carbon dioxide; BE, base excess; TCO2, total carbon dioxide; GCS, Glasgow Coma Scale; RRT, Renal Replacement Therapy.

Figure S1. The distribution of missing data in the original dataset (N = 24,272). A: Barplot illustrating missing proportions stratified by each feature; B: Heatmap revealing missing data stratified by each patient.

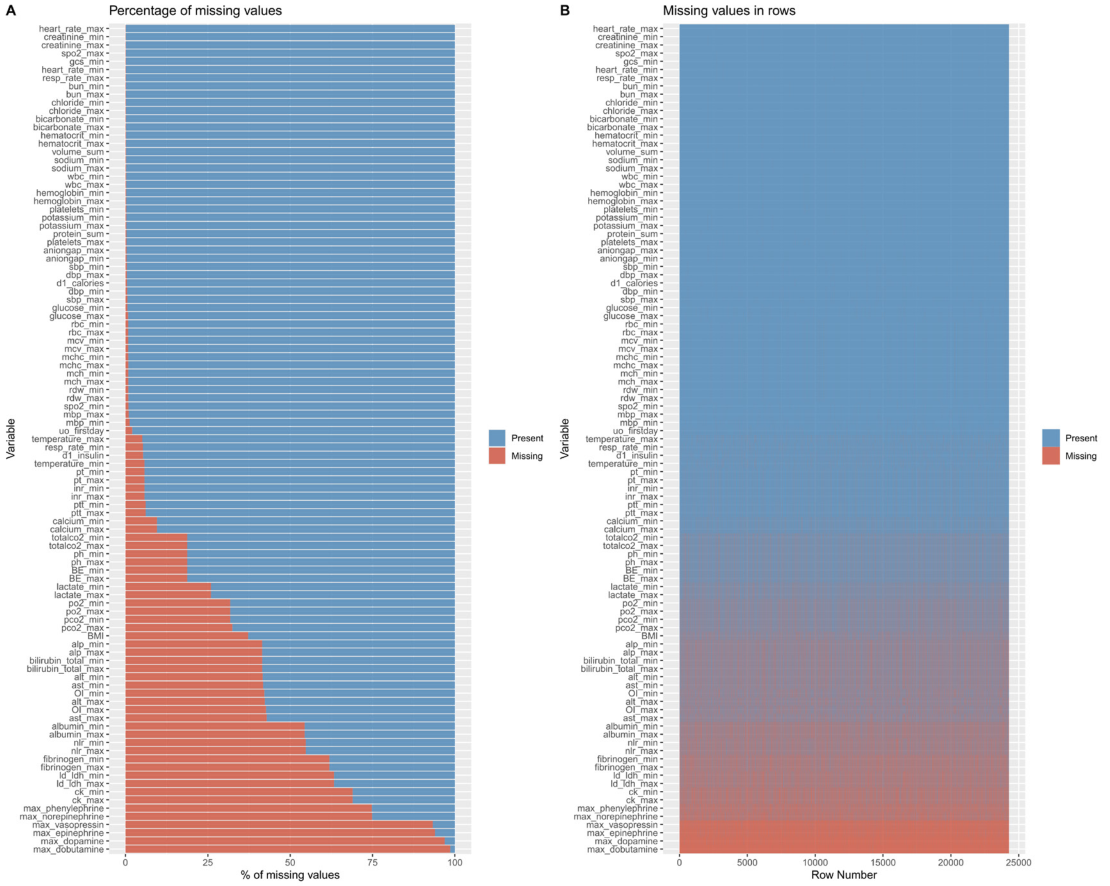

Blue stood for present values and red for absent values.

Figure S2. The distribution of original and imputed data points from features with over 40% missingness. A: Maximum AST; B: Minimum AST; C: Maximum ALT; D: Minimum ALT; E: Maximum total bilirubin; F: Minimum total bilirubin; G: Maximum ALP; H: Minimum ALP.

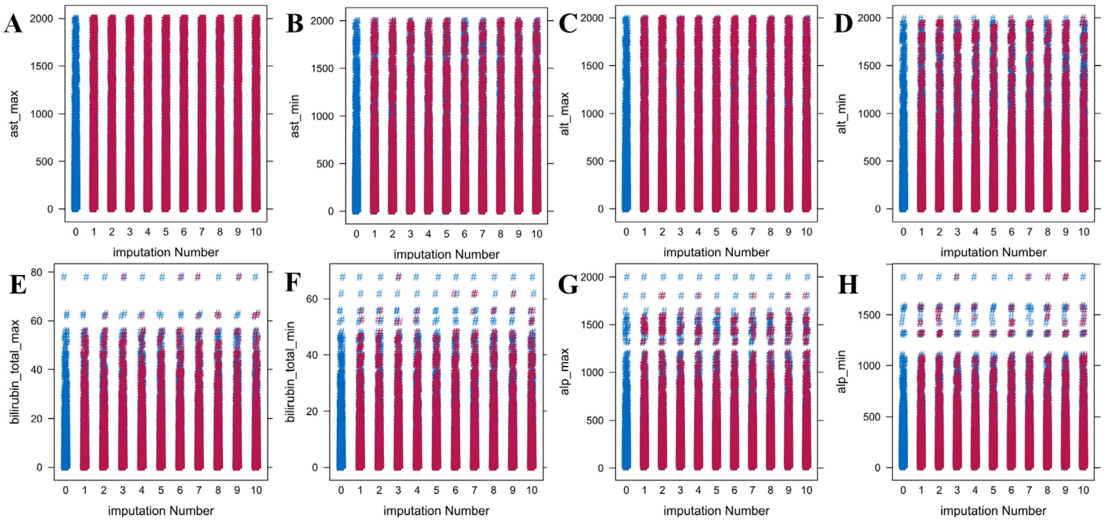

Blue spots represented pre-existing data and red spots represented imputed data. AST, aspartate transaminase; ALT, alanine transaminase; ALP, alkaline phosphatase.

Figure S3. Feature importance rankings in the "Whole" XGBoost model (top 20 features included).

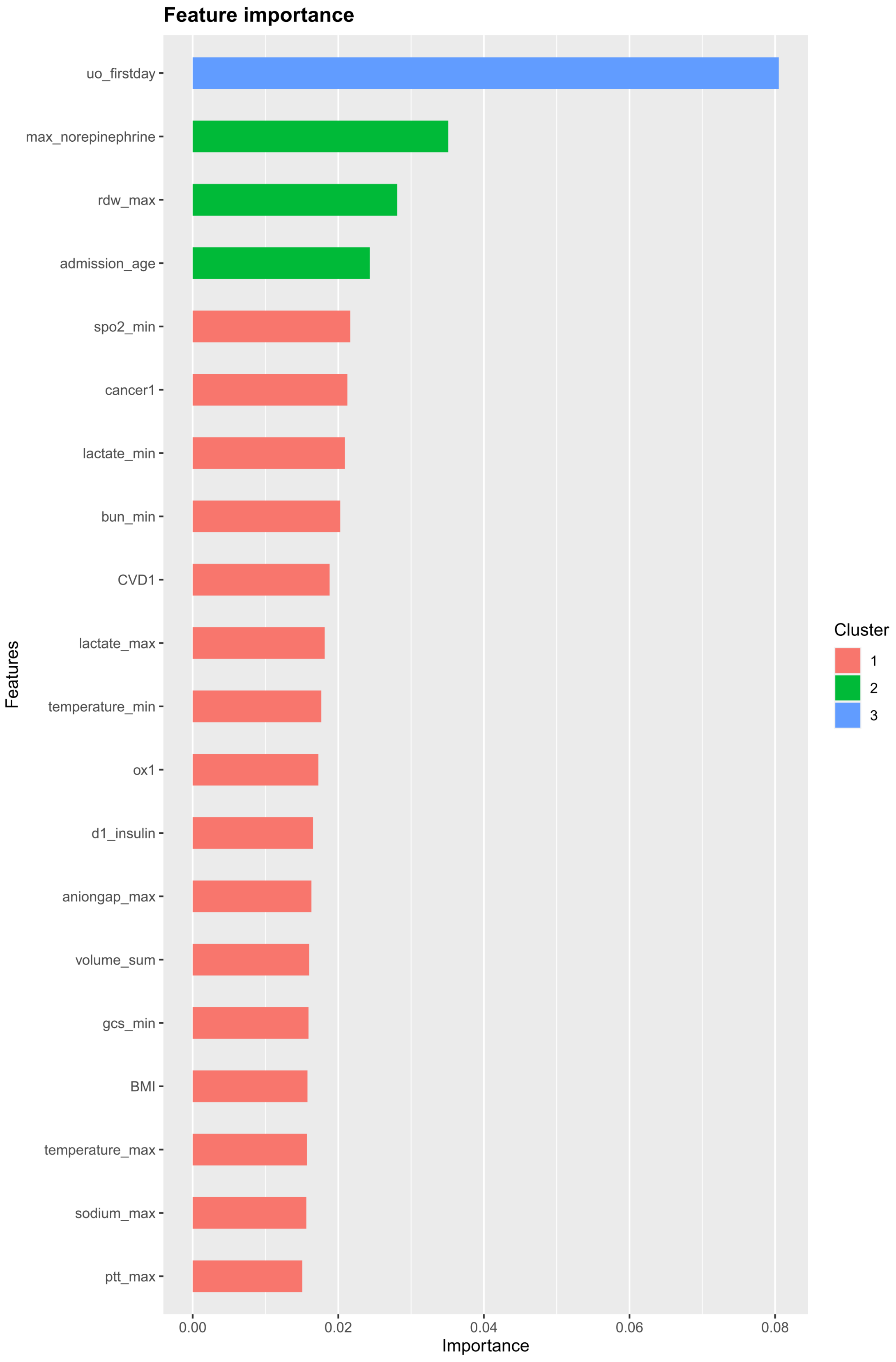

UO, urine output; RDW, red blood cell distribution width; SpO2, peripheral oxygen saturation; BUN, blood urea nitrogen; CVD, Cerebrovascular Disorder; OX, supplement oxygen; GCS, Glasgow Coma Scale; BMI, body mass index; PTT, partial

thromboplastin time.
